# Guided Imagery and Music modulates neural circuits in mood disorders: a systematic review of mechanisms and clinical efficacy

**DOI:** 10.3389/fpsyg.2026.1739840

**Published:** 2026-06-24

**Authors:** Tianyi Wang, Xin Zhao, Han Wang, Ying Li, Qitong Jiang, Yuan Feng, Gang Wang, Jing Du

**Affiliations:** 1 The National Clinical Research Center for Mental Disorders and Beijing Key Laboratory of Mental Disorders, Beijing Anding Hospital, Capital Medical University, Beijing, China; 2 Advanced Innovation Center for Human Brain Protection, Capital Medical University, Beijing, China

**Keywords:** emotional cognition, Guided Imagery and Music, mood disorders, neural circuits, neuroplasticity, non-pharmacological intervention, reward system

## Abstract

**Background:**

Guided Imagery and Music (GIM), as an emerging therapeutic approach, is widely applied in the treatment of Mood disorders (MDs). However, its efficacy and underlying neural mechanisms remain unclear at present. MDs represent one of the leading causes of disability worldwide. Conventional pharmacological treatments, which primarily target single biological pathways, are frequently limited by side effects and demonstrate inconsistent therapeutic efficacy. GIM is a multimodal psychotherapeutic intervention that utilizes music-evoked imagery to facilitate emotional and cognitive processing. By engaging cognitive and emotional processing networks, GIM is regarded as one of the approaches for neuropsychological rehabilitation in mood disorders. However, the underlying neural circuit mechanisms of this approach remain insufficiently systematized and warrant further investigation.

**Methods:**

We conducted a systematic review following PRISMA guidelines. A comprehensive literature search was performed in PubMed and Web of Science (2000–2024) to examine the neural mechanisms and clinical efficacy of GIM for MDs, with emphasis on randomized controlled trials (RCTs) and neuroimaging studies.

**Results:**

GIM modulates four key neural circuits implicated in MDs: (1) regulates amygdala hyperactivity while enhancing prefrontal-amygdala connectivity; (2) promotes hippocampal neuroplasticity via auditory-driven theta synchronization; (3) reorganizes prefrontal network coordination by strengthening dorsolateral prefrontal connectivity and reducing ventromedial default mode network (DMN) hyperconnectivity; and (4) activates the nucleus accumbens dopaminergic reward pathway to mitigate anhedonia. Clinically, GIM demonstrates significant efficacy in alleviating depressive symptoms, improving cognitive function, and reducing somatic complaints across diverse populations.

**Conclusion:**

This study proposes an integrated neural circuit-targeted model for GIM in MDs, bridging multimodal neural modulation with clinical outcomes. The findings establish a neuroscientific basis for GIM as a promising non-pharmacological intervention and support its integration into standard mood disorder treatment protocols. This review presents an integrative neural circuit framework for GIM in MDs, synthesizing existing neuroimaging and clinical evidence, and laying the groundwork for future mechanistic and clinical research.

## Highlights

- We propose a neural circuit-targeted model for Guided Imagery and Music (GIM) in treating Mood Disorders (MDs), which bridges multimodal neuromodulation mechanisms with clinical efficacy.
- GIM exerts dynamic modulation on key brain networks implicated in MDs, including the amygdala-centered emotion networks, hippocampal memory systems, prefrontal cognitive control circuits, and nucleus accumbens (NAc) reward pathways.
- Clinical evidence demonstrates that Guided Imagery and Music (GIM) exhibits cross-diagnostic therapeutic efficacy, showing significant improvements in mood symptoms, cognitive dysfunction, and somatic complaints among clinical populations with mood disorders, including Major Depressive Disorder (MDD) and Bipolar Disorder (BD).
- As an emerging non-pharmacological intervention, GIM shows substantial potential for enhancing brain function in MDs and offers a promising integrative approach for personalized treatment in psychiatry.

## 1 Introduction

Mood disorders (MDs) are a category of emotional disorders characterized by high suicide risk and prevalence, representing a disabling mental illness. Pharmacological treatment has long been the core intervention strategy for managing these conditions (Haregu et al., 2023). While conventional medications may alleviate certain symptoms, they frequently entail significant adverse effects, demonstrate variable efficacy, and lack the capacity for personalized adaptation to each individual’s unique experience with MDs. Furthermore, these pharmacological approaches typically target isolated neurotransmitter systems (e.g., serotonin or norepinephrine), thereby failing to address the complex neural circuit dysregulation and regional brain dysfunction characteristic of MDs. This therapeutic gap has spurred growing interest in non-pharmacological interventions capable of multi-target modulation.

Emerging evidence highlights the therapeutic potential of non-pharmacological modalities such as mindfulness meditation and transcranial magnetic stimulation (TMS) in modulating neural circuits and ameliorating psychiatric symptoms. Notably, these mechanisms are particularly relevant to Guided Imagery and Music (GIM), an innovative approach that harnesses multimodal sensory stimulation to target emotion- and cognition-associated brain regions (Susan and Eden, 2023; Reangsing et al., 2024). In addition to clinical populations, experimental studies on healthy adults have also demonstrated that music can significantly enhance emotional arousal and improve cognitive functions such as attention and executive control (Giannouli et al., 2024). These findings underscore the dual role of music as both an emotion regulator and a cognitive enhancer, supporting its broader applicability in mental health and neurocognitive optimization.

As a powerful multisensory medium, music demonstrates unique efficacy in activating neural substrates of emotion processing and memory consolidation (Jiang and Zhou, 2007). When combined with guided imagination—a potent modality for inner world expression—this synergy forms the foundation of GIM.

Developed in the 1970s by pioneering music therapist Helen Bonny, GIM distinguishes itself from conventional music therapy approaches through its distinctive methodology. Unlike passive listening or active improvisation techniques, GIM employs carefully curated musical selections to stimulate the auditory and emotional systems while guiding individuals through structured imagery experiences in a relaxed state. This innovative combination of music-evoked imagery facilitates neural activation in key brain regions, enabling individuals to reprocess negative emotional content and achieve enhanced cognitive-emotional integration. This review bridges the connection between emotional experiences and neural circuit changes in GIM, highlighting its value as an intervention approach grounded in neuropsychological theory.

It is noteworthy that an individual’s response to music therapy (including GIM) is not solely determined by biological mechanisms but is significantly influenced by their sociocultural background. The role, acceptability, and types of music (such as classical music and traditional ethnic music) in therapy vary markedly across different cultural contexts, constituting important situational factors in treatment. A study of young Greeks revealed that while most acknowledged the positive effects of music on improving mood and cognition, their specific attitudes, preferred treatment approaches (such as a preference for passive listening), and judgments regarding the applicability of music to particular conditions (such as cardiovascular diseases and depression) all reflected underlying cultural perceptions (Giannouli, 2013). Therefore, in clinical practice, therapists should fully assess and respect participants’ cultural backgrounds and musical preferences, which is crucial for establishing a therapeutic alliance, selecting appropriate musical materials, and ultimately enhancing the efficacy of interventions such as GIM.

### 1.1 Theoretical basis and intervention protocol of GIM

#### 1.1.1 Theoretical foundation

GIM is grounded in humanistic principles while integrating psychodynamic and neuroplasticity theories. Rooted in Rogers’ person-centered approach, GIM employs music to induce a relaxed state and establish a safe therapeutic environment. This facilitates inner imagery, thereby fostering self-awareness and emotional expression (Rusu, 2019). From a psychodynamic perspective, GIM leverages primary process thinking to activate subconscious conflict resolution, enabling individuals to explore repressed emotions and achieve psychological release (Lawes, 2020). Concurrently, neuroplasticity theory posits that music and imagery can restructure neural networks, particularly the default mode network (DMN) and salience network (SN), enhancing emotional regulation and self-healing through synaptic reorganization (Koelsch et al., 2021; Su Lin, 2021). In summary, GIM represents a multimodal therapeutic approach that is widely utilized in music therapy, combining psychological and neuroscientific frameworks to promote holistic healing.

#### 1.1.2 The process of GIM treatment

The therapeutic process of GIM typically comprises five core stages: prelude, relaxation, focus/theme development, Music and Imagery Experience (core), and finale (Bonny and Su, 2021). These stages are seamlessly interconnected, working synergistically to facilitate therapeutic outcomes.

1. **Prelude:** The therapist establishes a safe and trusting therapeutic relationship with the client. This stage involves understanding the patient’s life experiences, assessing their needs, collaboratively setting therapeutic goals, and reaching a mutual agreement on the treatment approach.
2. **Relaxation:** The patient adopts a comfortable posture and follows the therapist’s guidance to gradually enter a relaxed state, reducing physiological arousal. The objective is to minimize physical tension, facilitate receptivity to deeper states of consciousness, and cultivate present-moment awareness—laying the groundwork for subsequent imagery exploration.
3. **Focus or theme:** The therapist introduces a specific imagery scenario—such as a place, person, event, or scene—as a starting point for free association within the musical experience. The patient embarks on a self-directed, music-guided journey, allowing spontaneous imagery to unfold.
4. **Music and imagery experience (core):** This is the central phase of GIM. While listening to the music, the patient verbally describes or visually represents (e.g., through drawing) the emerging imagery. The therapist encourages nonjudgmental awareness of present-moment feelings, fostering deeper integration between emotion and cognition.
5. **Finale:** Following the music, the patient reflects on the imagery and emotions experienced. The therapist guides the patient in organizing the sequence of images and feelings, validating key insights, and connecting them to daily life. This process consolidates therapeutic gains and enhances the transfer of benefits into real-world contexts.

To delineate the unique therapeutic position of GIM, Table 1 provides a systematic comparison of its core methodologies, underlying mechanisms, and targeted neural circuits with other established music therapy approaches. The comparative analysis reveals that GIM’s distinctive feature resides in its depth-oriented, imagery-driven approach that synergistically combines psychodynamic processing with comprehensive neural circuit modulation. This dual mechanism—facilitating profound psychological exploration while simultaneously engaging multiple neural networks—establishes GIM as the foundation for its neural circuit-targeted therapeutic model in the treatment of mood disorders (MDs). **GIM Empirical:** Findings from studies explicitly using the Bonny Method of Guided Imagery and Music (GIM) or closely adapted protocols. **Music Therapy Evidence:** Findings from other music-based interventions (e.g., receptive music therapy, improvisation, music-assisted relaxation), which inform the general neurobiological context for music-based therapy. **Hypothesized Mechanism:** Mechanisms inferred from affective neuroscience or general music perception literature, considered plausible for GIM but not yet directly tested within GIM protocols.

**Table 1:** Comparing Guided Imagery and Music (GIM) with other common music therapy approaches for depression.

Music therapy approaches	Core approach	Therapist’s role	Patient’s action	Primary therapeutic mechanism	Targeted neural circuits	Music therapy evidence	Citation
Receptive music therapy	Structured listening to pre-composed music	Selector (of music)	Listening reflecting	Mood induction, emotional resonance	Limbic system, auditory cortex	Non-GIM music interventions	Delpech et al. (2021) and Ferdiana et al. (2024)
Active improvisational music therapy	Creating music in the moment with instruments/voice	Container (for musical expression)	Playing Improvising Interacting	Non-verbal communication, Expression, Interpersonal connection	Frontolimbic circuits	Active music-making studies	Erkkilä et al. (2019)
Music-assisted relaxation	Use music as a relaxation technique	Instructor (of relaxation protocol)	Following Relaxing	Autonomic nervous system regulation, Stress reduction	Parasympathetic system	Music and relaxation studies	Cordoba-Silva et al. (2024)
Guided imagery and music (GIM)	Bonny Method; Music-evoked imagery for exploratory psychotherapy	Guide facilitator (of imagery process) connection	Describing personal emotional and cognitive experiences	Multimodal integration: Auditory stimulation, Imagery, Emotional reprocessing, Therapeutic alliance	Amygdala-PFC connectivity, Hippocampal plasticity, NAc reward pathway	Direct GIM studies & Hypothesized Mechanism	Chou and Lin (2006), Lin et al. (2010), and Lee et al. (2016)

### 1.2 Application of music as a non-pharmacological intervention

Music, as a cross-cultural and multimodal non-pharmacological intervention, demonstrates broad application prospects in the field of mental health. In recent years, music-based psychotherapeutic approaches have been gradually integrated into clinical practice for MDs, with their mechanisms of action involving the modulation of neuroplasticity and the coordinated improvement of emotional processing and cognitive function. Unlike the single-target approach of traditional pharmacological treatments, music interventions achieve coordinated regulation across multiple brain regions and systems through the integration of auditory, emotional, and cognitive processes, thereby providing an effective avenue for the treatment of mood disorders.

Existing research indicates that music interventions can significantly influence neural networks associated with emotion regulation, including prefrontal-limbic connectivity, modulation of the default mode network (DMN), and activation of the reward system (Gold et al., 2023). Classical music and traditional ethnic music, characterized by their structural stability and rich emotional expression, are frequently employed to alleviate anxiety, enhance emotional stability, and facilitate relaxation and emotional expression in clinical settings (Jiao, 2025). Additionally, music can mitigate somatic symptoms associated with mood disorders by regulating autonomic nervous system activity (such as heart rate variability) and neuroendocrine responses.

In terms of treatment modalities, music interventions can be categorized into receptive music therapy, active improvisation, music-assisted relaxation, and guided imagery and music (GIM)—the focus of this paper—among others. These methods are applicable not only to depressive and anxiety disorders but also show significant potential in post-traumatic stress disorder (PTSD), psychosomatic diseases, and neurological rehabilitation. Notably, traditional ethnic music, due to its cultural affinity and emotional resonance, often enhances patient acceptance and participation in cross-cultural clinical environments (Shimada et al., 2021).

Overall, music-based non-pharmacological interventions, characterized by their high safety, ease of implementation, and strong individual adaptability, are gradually becoming an integral part of comprehensive psychiatric treatment systems. Looking ahead, with the advancement of neuroscience research and the integration of digital technologies, music interventions are expected to further promote personalized and precision treatment, offering more comprehensive rehabilitation support for patients with mood disorders.

Despite the established clinical procedures and theoretical foundations of GIM, a systematic framework elucidating how it modulates the dysfunctional neural circuits characteristic of MDs remains absent. This systematic review therefore aims to develop an integrative neural circuit-targeted model for GIM in MDs by synthesizing existing neuroimaging evidence. Evaluate clinical efficacy and transdiagnostic potential across diverse mood disorder populations and symptom domains.

## 2 Methods of literature search

### 2.1 Literature search strategy

A systematic literature search with a narrative-oriented synthesis was conducted to identify studies examining the effects of Guided Imagery and Music (GIM) on neural circuits associated with mood disorders. Electronic searches were performed in PubMed and Web of Science from January 2000 to March 2024. Search terms were organized into conceptual domains related to music-based interventions and affective disorders, including combinations of “Guided Imagery and Music,” “music therapy,” “music,” “mood disorders,” “neuroplasticity,” “reward system,” and “non-pharmacological intervention.”

### 2.2 Eligibility criteria

Studies were eligible for inclusion if they involved Guided Imagery and Music (GIM) or closely related interventions incorporating therapist-guided imagery combined with music listening, and if they reported clinical, behavioral, or neurobiological outcomes relevant to mood disorders. Both clinical intervention studies and mechanistic investigations were considered.

With respect to population characteristics, studies including adult or mixed-age samples were eligible. Both clinically diagnosed populations and individuals presenting elevated mood symptoms assessed using validated psychometric instruments were included. Given the heterogeneity of diagnostic approaches across the literature, no specific diagnostic classification system (e.g., DSM or ICD criteria) was required for inclusion, provided that mood-related outcomes were clearly defined and reported.

Studies were excluded if they focused solely on passive music listening, background music exposure, music education or training, or other non-therapeutic uses of music. In addition, non-peer-reviewed publications, conference abstracts, dissertations, and non-English articles were excluded.

### 2.3 Study selection process

The initial search yielded 551 records. After removal of duplicate and clearly irrelevant records (*n* = 102), 449 articles were screened based on titles and abstracts. Of these, 222 full-text articles were assessed for eligibility. Thirty-two full-text articles were excluded due to insufficient methodological detail or failure to meet the inclusion criteria. A total of 190 studies were ultimately included in the narrative synthesis.

### 2.4 Quality assessment, risk of bias, and evidence weighting

Study quality and risk of bias were assessed according to study design. Randomized controlled trials (RCTs) were appraised using the Cochrane Risk of Bias 2 (RoB 2) tool, focusing on randomization procedures, blinding (where applicable), and completeness of outcome reporting. Non-randomized and observational studies were evaluated using key domains of the ROBINS-I framework, including confounding and participant selection.

Mechanistic and neuroimaging studies were assessed qualitatively in terms of sample size adequacy, methodological transparency, and analytical rigor, rather than through formal risk-of-bias scoring. In the narrative synthesis, mechanistic neuroimaging studies were considered primarily for elucidating neurobiological mechanisms and theoretical frameworks and were not weighted equivalently to clinical trials when interpreting therapeutic efficacy. Greater evidentiary weight was assigned to clinical trials in drawing conclusions regarding clinical relevance and treatment effects.

### 2.5 Reporting framework

**Figure 1:**
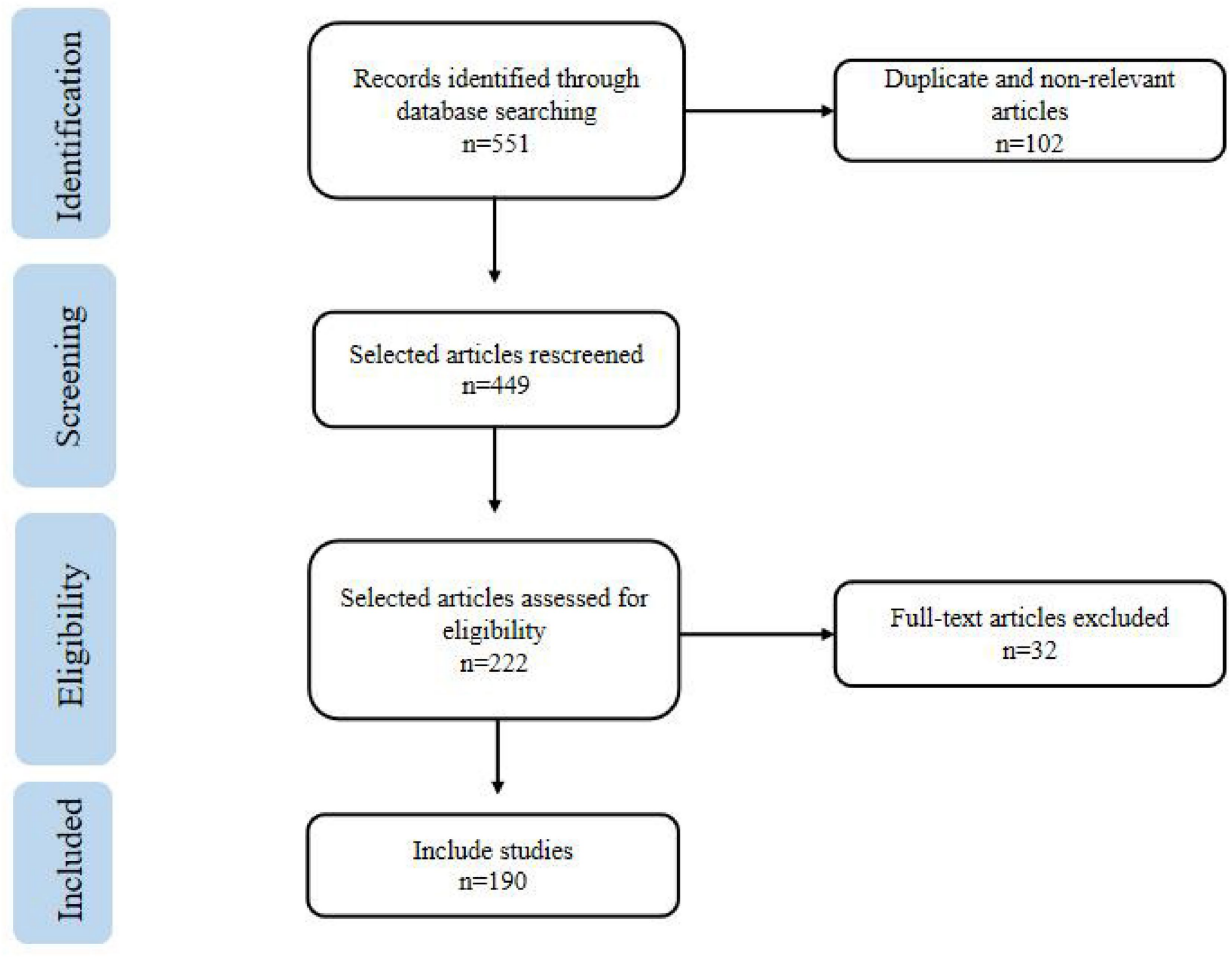
PRISMA flow diagram of study selection for the systematic review on Guided Imagery and Music (GIM) and mood disorders. This flowchart details the identification, screening, eligibility, and inclusion process for studies examining GIM and related music interventions. The included studies form the evidence base for this review, which is categorized into three levels: GIM empirical (direct GIM studies), music therapy evidence (broader music intervention studies), and hypothesized mechanisms (inferences from affective neuroscience).

The literature search and study selection process adhered to the Preferred Reporting Items for Systematic Reviews and Meta-Analyses (PRISMA) 2020 reporting guidelines, with adaptations appropriate for a narrative-oriented synthesis. A PRISMA flow diagram summarizing the study selection process is presented in Figure 1.

### 2.6 Rationale for integrating music and neuroscience

When conducting a systematic review of the neural mechanisms underlying GIM, we adopted an integrative evidence synthesis strategy. Specifically, in addition to studies directly employing the Bonny Method of GIM, we strategically incorporated research findings from a broader range of music therapies (such as receptive music therapy and music-assisted relaxation therapy) as well as the field of basic affective neuroscience. This integration provides a theoretical basis for the mechanism rationality and biological foundation of GIM’s multimodal intervention format—which synergizes auditory, imaginative, and therapeutic relationship elements—particularly in areas such as music-induced emotions, neural plasticity, and reward processing.

Moreover, given the current relative scarcity of high-quality neuroimaging literature directly targeting GIM, incorporating relevant and well-researched music intervention modalities along with foundational neuroscientific evidence helps establish a more stable neural circuitry model. By clearly labeling the sources of evidence, we ensure that direct evidence, indirect support, and theoretical inferences can be distinguished during the comprehensive narrative process, thereby enhancing the scientific rigor of our interpretations and avoiding overgeneralization (Figure 1 and Supplementary materials.)

## 3 Results

### 3.1 Mechanisms of action of GIM target relevant brain regions in MDs

Mood disorders are complex conditions rooted in abnormalities across multiple brain regions within the emotional regulation circuitry. Dysfunctional neural networks underlie these disorders, resulting in impaired emotional regulation, cognitive deficits, and abnormal reward processing. The following discussion examines the pathophysiological mechanisms involving four key brain regions: the amygdala, hippocampus, prefrontal cortex (PFC), and nucleus accumbens (NAc).

#### 3.1.1 Amygdala: abnormalities in negative emotion processing and functional connectivity

The amygdala is a core brain region critically involved in emotional processing, playing a key role in the formation and regulation of emotional responses, particularly in heightened reactivity to negative stimuli (Bradt and Teague, 2018). Neuroimaging studies have demonstrated that patients with MDs exhibit hyperactivation of the amygdala in response to negative cognitive stimuli (Koelsch, 2020). Resting-state functional magnetic resonance imaging (fMRI) further reveals that weakened functional connectivity between the amygdala and dorsolateral prefrontal cortex (dlPFC) is closely associated with impaired emotional inhibition, leading to persistent negative emotions and exacerbated depressive symptoms (Berboth and Morawetz, 2021). Additionally, reduced gray matter volume in the amygdala has been linked to decreased brain-derived neurotrophic factor (BDNF) levels, showing a significant negative correlation with disease severity, which suggests its potential as a biomarker for MDs (Bradt et al., 2016). Dysregulation of the dorsomedial prefrontal cortex–basolateral amygdala–ventral hippocampus (dmPFC–BLA–vHPC) circuit is considered a central mechanism underlying emotion regulation deficits in MDs. Recent studies suggest that impaired GABAergic inhibition in the basolateral amygdala (BLA) and enhanced glutamatergic signaling in the central amygdala (CeA) contribute to the persistence of negative emotional states (Tye et al., 2011).

#### 3.1.2 Hippocampus: memory deficits and downregulation of neuroplasticity

The hippocampus plays a pivotal role in memory formation and auditory perception, particularly in the encoding and retrieval of emotional memories. It exhibits strong functional connectivity with the amygdala, NAc, and PFC—regions critically involved in memory processing and storage. Reduced hippocampal volume is a well-documented neural hallmark of MDs. Studies indicate that hippocampal gray matter density is 12% lower in MDs patients compared to healthy controls, showing a significant negative correlation with Hamilton Depression Rating Scale (HAMD-17) scores (Frodl et al., 2017). Chronic stress leads to decreased hippocampal neuroplasticity through inhibition of BDNF expression, reduction of dendritic spine density, and impaired neurogenesis, which in turn triggers memory deficits and impaired emotion regulation (Zeidman and Maguire, 2016). Furthermore, microglial activation releases pro-inflammatory cytokines (e.g., IL-6, TNF-*α*), which further inhibit hippocampal neurogenesis. Notably, anti-inflammatory treatments have been shown to restore hippocampal neuroplasticity (Pan et al., 2014). At the cellular level, synaptic plasticity changes primarily involve two mechanisms: long-term potentiation (LTP) and long-term depression (LTD). Downregulation of NMDA receptors in the hippocampal CA1 region reduces synaptic transmission efficiency, disrupting the LTP/LTD balance and exacerbating memory impairments in MDs patients (Ge et al., 2010; Koelsch et al., 2013).

#### 3.1.3 Prefrontal cortex: cognitive control deficits and functional remodeling

The PFC serves as a critical hub for executive function and emotional regulation. Patients with MDs exhibit reduced dlPFC activity and diminished ventromedial prefrontal cortex (vmPFC) functional connectivity, leading to impaired cognitive control and emotion dysregulation (Arnsten, 2009; Pizzagalli, 2011). Interestingly, music exposure during exercise enhances right auditory cortex–prefrontal connectivity and emotional arousal, promoting externally directed attention (Pan et al., 2019; Li et al., 2019). These prefrontal abnormalities exacerbate emotional–cognitive dysregulation, which the Triple Network Model (TNM) conceptualizes as an imbalance between the default mode network (DMN), salience network (SAL), and central executive network (CEN). Imbalances within this network underlie cognitive–emotional dysregulation (Bolton et al., 2020).

#### 3.1.4 Nucleus accumbens: dysregulation of the reward system and diminished dopaminergic signaling

The NAc serves as the central hub of the reward system, situated at the interface between the basal ganglia and limbic system. It plays crucial roles in addiction, motivated behavior, reward processing, and emotion regulation (Yan et al., 2023). In MDs, diminished dopamine signaling leads to anhedonia—the inability to experience pleasure—which represents a core symptom of the condition (Nestler and Carlezon, 2006). Research demonstrates that D2 receptor binding in the NAc is reduced by 25% in MDs patients compared to healthy controls, strongly correlating with pleasure deficits (Tye et al., 2013). Functional MRI studies reveal that decreased NAc signaling is associated with weakened PFC connectivity in MDD patients, resulting in reward system dysfunction and reduced motivated behaviors (Reybrouck and Brattico, 2015). The Reward Prediction Error Hypothesis (RPH), a fundamental concept in reinforcement learning theory, proposes that dopamine neurons encode prediction errors to guide adaptive behavior. This hypothesis further suggests that reduced dopamine release diminishes an individual’s responsiveness to positive stimuli, leading to decreased reward anticipation and exacerbation of depressive symptoms (Zweifel et al., 2009; Treadway et al., 2012).

In summary, the neural mechanisms underlying MDs involve functional abnormalities across multiple brain regions, including amygdala hyperactivation, decreased hippocampal neuroplasticity, imbalanced PFC network coordination, and NAc reward function dysregulation. These neural alterations not only impair emotion regulation and cognitive function but also offer potential therapeutic targets for GIM interventions. Future research should further investigate the interactive mechanisms among these brain regions to advance the development of precision therapies for MDs.

### 3.2 GIM affects the MDs-related brain regions

Building upon the pathophysiological abnormalities in key brain regions described above, GIM exerts its therapeutic effects by dynamically remodeling these dysfunctional circuits through multimodal neuromodulatory mechanisms. Specifically, GIM modulates the amygdala-based emotion network, hippocampal neuroplasticity, the prefrontal cognitive control network, and the NAc-mediated reward pathway (Koelsch, 2020). This multidimensional neuromodulation provides both therapeutic support for MDs and integrated insights into GIM’s multi-target mechanisms.

Neuroimaging evidence further demonstrates GIM-induced neural changes. Recent fMRI studies, which measure blood oxygen level-dependent (BOLD) signals to map brain activity, reveal that GIM activates neurorestorative processes and modulates stress response systems (Chand et al., 2022).

#### 3.2.1 Amygdala: dynamic balance of emotional networks

GIM modulates the amygdala—a key region for emotion and memory processing—through music’s emotional potency and rhythmic properties, dynamically regulating subregional activity and cross-network functional connectivity (Mori and Zatorre, 2024). Functional near-infrared spectroscopy (fNIRS) studies demonstrate that soothing music strengthens functional connectivity between the basolateral amygdala (BLA) and anterior cingulate cortex (ACC), facilitating positive emotional integration (Koelsch, 2020). Concurrently, inhibition of the central amygdala (CeA) reduces its overactivation, improving emotion regulation network coordination and attenuating negative emotional responses (Lv et al., 2024). Further research reveals that music-induced *α*–*γ* cross-frequency coupling optimizes oscillatory coherence in the amygdala–insula–NAc circuit, enhancing the synergy between emotional evaluation and reward processing, thereby alleviating emotional dysregulation in depression (Castro et al., 2020).

#### 3.2.2 Hippocampus: enhanced plasticity of the auditory system

GIM engages the hippocampal–parahippocampal gyrus and entorhinal cortex via complex auditory stimulation, promoting contextual memory reconsolidation (Han et al., 2023). This effect is particularly pronounced in the hippocampus, which is critically involved in memory formation and emotion regulation. Diffusion tensor imaging (DTI), an advanced MRI technique for mapping white matter microstructure, was employed in an 8-week intervention study. Results showed increased axial diffusivity (AD) in hippocampal white matter tracts, indicating microstructural axonal optimization (Wan and Schlaug, 2010). Moreover, synchronization of musical rhythms with hippocampal theta oscillations enhances dentate gyrus neurogenesis and functional connectivity within the hippocampal–default mode network. These mechanisms collectively improve self-referential processing and mitigate MDs-related memory impairments.

#### 3.2.3 Prefrontal cortex: remodeling of network synergy

Guided imagery and music exerts regulatory effects on emotional processing through the medial prefrontal cortex (mPFC), demonstrating significant involvement in both emotional and social-cognitive functioning. The intervention enhances cognitive control via the dlPFC while simultaneously optimizing functional connectivity between the vmPFC and the DMN (Lassner et al., 2024). Resting-state fMRI showed enhanced dlPFC–amygdala functional connectivity and weakened vmPFC–DMN connectivity after the intervention, suggesting an effective improvement in cognitive–emotional network integration (Pan et al., 2019). Furthermore, through predictive coding of musical tempo, GIM enhances reward anticipation responses in the prefrontal–striatal circuit, thereby optimizing motivational and behavioral regulation (Gold et al., 2023).

#### 3.2.4 Nucleus accumbens: targeted activation of dopaminergic pathways

Guided imagery and music selectively activates dopaminergic pathways in the NAc through specific musical elements. PET–CT imaging combines positron emission tomography (PET), which provides molecular-level functional and metabolic information, with computed tomography (CT), which offers precise anatomical localization. This hybrid imaging modality enables comprehensive tomographic imaging with high sensitivity, accuracy, and specificity. Research using this methodology has demonstrated that pleasurable musical stimulation increases dopamine D2 receptor binding in the NAc by 18%, a change significantly correlated with enhanced subjective pleasure experiences (Treadway et al., 2012). Furthermore, the phase synchronization between musical tempo and limbic system activity strengthens oscillatory coupling within the NAc–ventral tegmental area (VTA) circuit. This neural mechanism restores adaptive responsiveness in the reward system and alleviates core symptoms of MDs (Zweifel et al., 2009).

**Figure 2:**
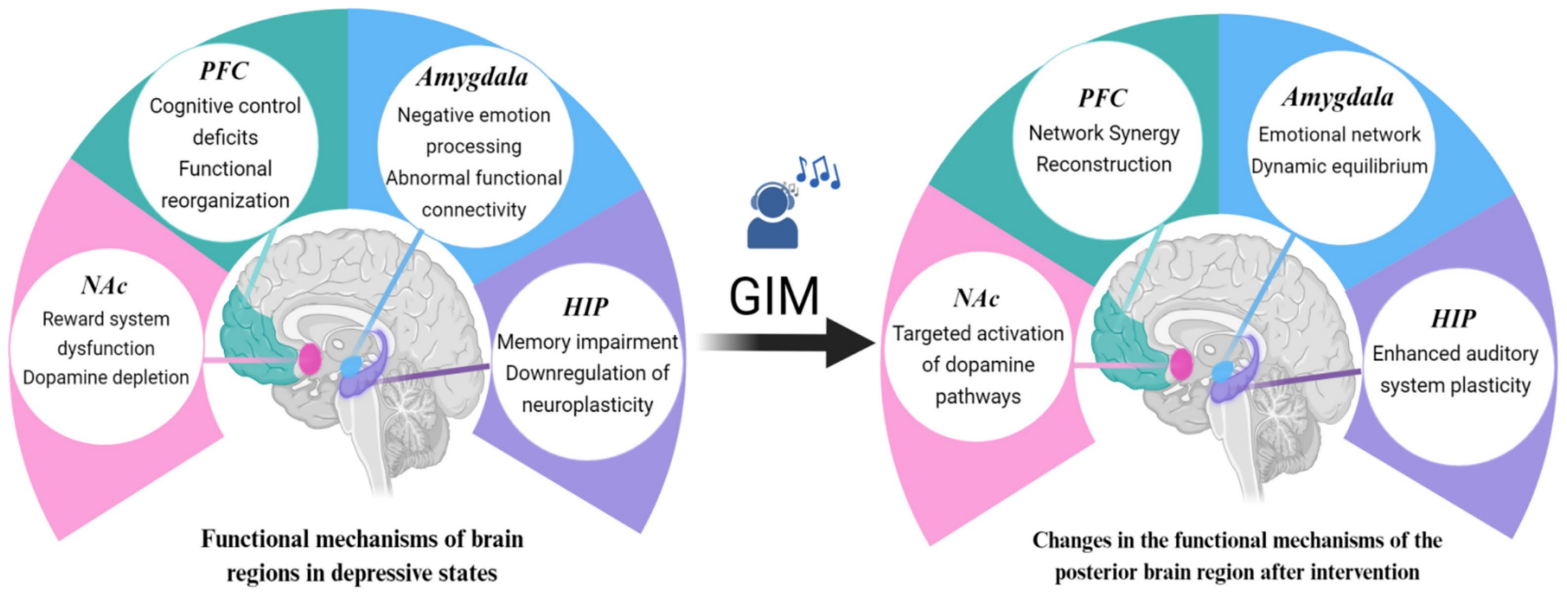
Proposed neurocircuitry model of GIM mechanisms in mood disorders.

In summary, GIM induces comprehensive remodeling of neural activity patterns in MDs-related brain regions through multiple neuromodulatory mechanisms, including enhanced functional connectivity and optimized oscillatory coupling (Figure 2). The intervention’s core therapeutic pathways encompass dynamic rebalancing of amygdala emotional networks, auditory-driven enhancement of hippocampal plasticity, synergistic reorganization of prefrontal cortical networks, and targeted activation of NAc reward pathways. These neurobiological effects, synthesized into an integrated circuit model, provide a scientific foundation for GIM’s efficacy as a non-pharmacological intervention for MDs. Future clinical applications could integrate real-time neurofeedback technology to further refine intervention precision, potentially offering an effective psychotherapeutic approach for MDs patients.

The left side shows the typical functional abnormalities of relevant brain regions in MDs patients. It focuses on: dysregulation of reward system in the NAc, weakening of dopamine signaling (reduction of D2 receptor binding rhythm); cognitive control deficits in the PFC, functional remodeling, etc. (weakening of dorsal lateral prefrontal activity); enhancement of amygdala’s negative emotion processing, functional connectivity abnormality (weakening of amygdala-prefrontal connectivity); memory deficits in the hippocampus, down-regulation of neural plasticity (reduction in volume, reduction in density of dendritic spines), and so on. After GIM system intervention, the right side is remodeling the function of the corresponding brain regions. Targeted activation of the dopamine pathway in the NAc (upraised D2 receptor binding rhythm); optimization of network synergies in the PFC (enhanced dlPFC-amygdala connectivity, weakened vmPFC-DMN connectivity); optimization of emotional network coordination in the amygdala (alpha-gamma cross-frequency coupling to optimize the consistency of loop oscillations); hippocampus enhancement of plasticity in the auditory system (optimization of white matter microstructures, activation of hippocampal-default network connectivity). In summary, GIM achieves multi-target intervention by enhancing functional connectivity, optimizing neural oscillatory coupling and other multimodal mechanisms (Created with BioRender.com).

### 3.3 Clinical application of musical therapy and GIM in improving MDD

As non-pharmacological interventions, both music therapy and GIM demonstrate multidimensional clinical value in treating MDs. However, compared to standard music therapy, GIM’s core advantage lies in its ability to form a multimodal, integrated intervention approach. This is achieved through emotional remodeling, cognitive function enhancement, and comprehensive assessment of somatic symptoms, thereby providing mood patients with personalized, multidimensional adjunctive treatment plans (Table 2).

**Table 2:** Summary of clinical trials investigating GIM for MDD across patient populations.

Evidence type	Study design	Participants	Intervention	Outcomes (vs. control)	Neural mechanisms evidence	Diagnostic category	Effect size (estimate)	Citation
GIM empirical	RCT (Pilot)	Breast/gynecologic cancer patients with MDD (n = 62)	8 sessions (60 min, 2/week)	HADS-D:↓ 42.6%*; Response rate: 83.3% (OR = 4.1)	↑ dlPFC-amygdala FC (*r* = 0.71, *p* = 0.003)	MDD with medical comorbidity	OR = 4.1; Large effect	Papanikolaou et al. (2023)
GIM Empirical	RCT	Women with sexual dysfunction (*n* = 72)	6 weeks (60 min,2/week)	FSFI:↑42%*(*d* = 1.1)	↑18%NAc dopamine D2 receptor binding (PET-CT)	Depression with sexual dysfunction	Cohen’s *d* = 1.1; Large effect	Mohammadi et al. (2023)
GIM empirical	RCT	IBD patients in remission (*n* = 43)	8 sessions (2/h)	HADS:↓3.7%; HRV:↑42%; IBD-QOL:↑22.5**	↑Limbic system activity (fMRI: *r* = 0.56, *p* < 0.01)	Depression with medical comorbidity	Small-to-moderate effect (% change)	March-Luján et al. (2021)
GIM empirical	Non-randomized trial/Observational	Danish workers on sick leave (*n* = 20)	9 weeks	83% of the participants had returned to work	None	Work-related distress/Adjustment disorder	83% functional recovery	Beck et al. (2015)
GIM empirical	RCT	Adults with basal cell carcinoma and squamous cell carcinoma who received surgical resection (*n* = 155)	least 4 days before their surgery	STAI↓*	None	Pre-surgical anxiety	Significant reduction (*p* < 0.05)	Alam et al. (2016)

Table provides a structured summary of GIM clinical trials, now including estimated effect sizes by diagnostic category and qualitative ratings of evidence strength to facilitate comparisons across different populations and outcomes. GIM Empirical: Findings from studies explicitly using the Bonny Method of Guided Imagery and Music (GIM) or closely adapted protocols. Study Design Legend: RCT = Randomized Controlled Trial; Pilot = Pilot Randomized Controlled Trial (typically smaller sample, feasibility-focused); Non-randomized/Observational = Study without random allocation to groups. Diagnostic Category reflects the primary mood-related condition of the study population. Effect Size Estimates: OR (Odds Ratio) > 1 indicates favorable outcome for GIM; Cohen’s *d*: 0.2 = small, 0.5 = medium, 0.8 = large. HADS-D = Hospital Anxiety and Depression Scale-Depression; FSFI = Female Sexual Function Index; HRV = Heart Rate Variability; FC = Functional Connectivity; NAc = Nucleus Accumbens; dlPFC = dlPFC, STAI=State–Trait Anxiety Inventory. Statistics: *p < 0.05, **p < 0.01, ***p < 0.001; Cohen’s *d* for effect size; OR = Odds Ratio. Neural evidence derived from fMRI, PET-CT, and fNIRS data in Section 4.

#### 3.3.1 Developing positive emotions and relieving negative emotions

Music can evoke strong emotional responses, including pleasure, sadness, anger, and fear (Egermann and Reuben, 2020). As a non-verbal therapeutic modality, GIM harnesses this emotional power by facilitating emotional reorganization and insight through music and imagery. This process helps individuals identify, focus on, and become consciously aware of their emotional states, thereby alleviating negative emotions and fostering positive emotional development (Esteves and Conceição, 2022). Such heightened emotional awareness enhances positive affect and promotes deeper emotional processing and reflection. A study by Lin (2021) applied GIM to elderly depressed patients, measuring outcomes immediately post-intervention and at 4-week follow-up. Results demonstrated a 42.3% reduction in HAMD-17 scores compared to baseline (*p* < 0.001, Cohen’s *d* = 1.2), exceeding the minimal clinically important difference (MCID) threshold of 25%. These findings indicate that GIM significantly improves emotional experiences in patients with mood disorders. Beyond mood disorders, non-pharmacological interventions such as GIM also show therapeutic benefits in pain management among cancer survivors (Hikmat Hadoush et al., 2025). A randomized controlled trial (RCT) involving breast and gynecologic cancer patients found that GIM significantly reduced anxiety and MDD scores while enhancing overall emotional wellbeing (Papanikolaou et al., 2023). Collectively, these studies underscore GIM’s efficacy in mitigating negative emotions and promoting positive affective states across diverse clinical populations.

Regarding cognitive-emotional enhancement, GIM uses musical stimulation to elicit emotional responses while guiding individuals toward introspection and reflection. Research indicates that GIM not only alters emotional states but also improves emotional resolution (Vásquez-Rosati et al., 2019), suggesting that emotional distress can be restructured through guided affective imagery and reflective transformation. As evidenced, GIM supports emotional expression, inner exploration, and self-reflection by regulating affective processes, further validating its clinical utility in the treatment of MDs.

#### 3.3.2 Enhancement of cognitive function

GIM effectively alleviates cognitive symptoms in depressed patients, particularly in the domains of attention and memory. For individuals experiencing memory impairment, regular exposure to familiar music helps mitigate depressive symptoms by triggering emotional responses and enhancing memory encoding, thereby aiding memory restoration and improving quality of life (Leubner and Hinterberger, 2017; Raghavendra et al., 2022). Research indicates that GIM therapy induces significant neural activation in MDs patients during the recall and re-experiencing of autobiographical memories (Perilli, 2017), suggesting its role in facilitating memory retrieval and processing.

Regarding attention, GIM redirects focus away from negative emotions, elevates dopamine levels, alleviates low mood, and reduces distractibility caused by negative affect. This promotes a more positive mindset while enhancing attentional allocation and focus (Ramirez et al., 2015). Neuroplasticity—the brain’s ability to adapt its structure and function in response to environmental and experiential stimuli—is further augmented by GIM (Grocke, 2005). Through multisensory integration and emotional expression, GIM fosters structural and functional brain changes that enhance imagination and creativity (Reybrouck and Brattico, 2015). Collectively, these findings support GIM’s therapeutic potential and provide a robust scientific foundation for cognitive interventions in mood disorders.

#### 3.3.3 Improvement of somatic symptoms

Emerging evidence highlights GIM’s efficacy in improving sleep among patients with mood disorders. Therapists guide patients into a relaxed state, using music to facilitate inner exploration, thereby releasing negative emotions, reducing tension, and alleviating MDs symptoms—ultimately improving sleep quality (Huang, 2024). A study by Zhu et al. (2016) randomized 100 depressed patients (mean age: 40 years) with sleep disorders into two groups: a control group receiving conventional pharmacotherapy (*n* = 50) and an experimental group combining pharmacotherapy with music-based intervention (*n* = 50). Post-intervention analysis revealed significant reductions in total Pittsburgh Sleep Quality Index (PSQI) scores, including improvements across five dimensions: subjective sleep quality, sleep latency, sleep efficiency, sleep disturbances, and hypnotic medication use. These results confirm GIM’s role in ameliorating sleep disorders in patients with MDD. Further studies demonstrate that GIM’s antidepressant effects extend to elderly populations, with sleep quality serving as a key mediator. Among older adults with MDD and insomnia, GIM significantly reduced depressive symptoms while enhancing sleep quality and treatment satisfaction compared to other age groups (Loewy, 2020). Thus, for elderly MDD patients with insomnia, psychological interventions such as GIM—when combined with pharmacotherapy—yield clinically meaningful benefits.

As an adjunctive therapy, GIM demonstrates substantial efficacy in improving mood, cognitive function, and somatic symptoms in MDD patients. With continued research, GIM is poised to become an integral component of MDD treatment protocols.

### 3.4 Clinical application of GIM in other mood disorders

#### 3.4.1 Anxiety disorders (AD)

Anxiety disorders (AD) are among the most common mental health conditions, typically emerging in youth or adulthood and characterized by excessive worry, fear, nervousness, and dread (Penninx et al., 2021). These symptoms often cause significant distress in daily functioning. A landmark 2021 study found that for perinatal women, GIM promotes physical and mental relaxation, reduces anxiety symptoms, facilitates self-exploration and emotional release, and exerts favorable effects on fetal, preterm, and neonatal development (Sanfilippo et al., 2021; Shimada et al., 2021). For pregnant women in labor, GIM effectively lowers anxiety by redirecting attention away from discomfort or pain. Through guided visualization of a healthy pregnancy, fetal safety, and a positive birthing experience—combined with breathing techniques, muscle relaxation, and multisensory (visual, auditory, olfactory) stimulation—GIM elicits beneficial emotional responses (Ferdiana, 2024). Beyond perinatal care, GIM also benefits patients undergoing high-risk surgeries. Ela Susilawati et al. 2019 and Mordeno et al. (2016) used quota sampling to divide 38 anxious preoperative patients into an experimental group (n = 19, receiving nurse-led GIM) and a control group (n = 19, no intervention). Results demonstrated significantly lower preoperative anxiety in the GIM group. These findings suggest that GIM mitigates anxiety by disrupting threat-focused attention, thereby reducing fear responses—particularly in maternal populations.

#### 3.4.2 Post-traumatic stress disorder (PTSD)

Post-traumatic stress disorder (PTSD) is a complex psychiatric condition triggered by severe psychological trauma, with core symptoms including re-experiencing, avoidance, hypervigilance, and emotional numbness. Its prevalence rises with increasing exposure to traumatic events (Mordeno et al., 2016; Merians et al., 2022). Research indicates that GIM aids PTSD recovery by providing a nonverbal, safe avenue for emotional expression, traumatic memory processing, and cognitive-emotional rehabilitation (Fraile et al., 2023). Through therapist-guided imagery within a musical framework, GIM introduces positive affective states into traumatic recollections, helps patients reconstruct negative self-perceptions, and fosters emotional release (Richards, 2016; Herff et al., 2021).

#### 3.4.3 Neurorehabilitation applications

Guided imagery and music also shows promise in neurorehabilitation. Studies report broad benefits for stroke patients, including improvements in motor function, speech, perception, and cognitive-emotional processing. By enhancing motor cortex excitability via auditory input, GIM promotes functional brain reorganization and sensorimotor integration, accelerating post-stroke recovery (Lin and Zhou, 2024).

As an adjunct or alternative to conventional treatments, GIM enables patients to safely reconnect with memories, emotions, and bodily sensations. Its demonstrated efficacy in MDD, anxiety disorders, PTSD, and other mood disorders highlights considerable clinical potential. With ongoing research, GIM may become a cornerstone therapy for these conditions.

## 4 Discussion

This study presents a synthesized neural circuit-targeting model for GIM in MDs, integrating neuroimaging and clinical evidence. However, several limitations should be noted. First, there remains a limited number of high-quality randomized controlled trials (RCTs) specifically investigating GIM for MDs, particularly for bipolar disorder (BD). Second, some existing studies feature small sample sizes, potentially restricting the generalizability of findings. Additionally, GIM’s therapeutic process inherently incorporates subjective factors, such as musical preferences, individual imagery capacity, and therapist–patient rapport. While these elements facilitate personalized treatment, they complicate standardization and reproducibility in research.

It is noteworthy that GIM emerges as a promising non-pharmacological intervention for MDs, with accumulating evidence supporting its efficacy. Its therapeutic potential stems from its ability to modulate key neural circuits: the enhancement of prefrontal connectivity underpins improvements in cognitive control and executive function; hippocampal plasticity facilitates the reconsolidation of autobiographical memory; amygdala modulation aids in the reorientation of attentional resources away from negative bias; and NAc activation restores motivation and reward-based learning. Unlike conventional pharmacotherapy, GIM offers superior safety and flexibility, positioning it as a viable first-line adjunct therapy in primary care—an advantage of particular importance given global disparities in mental healthcare access.

Future research should prioritize large-scale, high-quality RCTs to further validate GIM’s efficacy relative to standard treatments. Combining GIM with neuromodulation techniques such as transcranial magnetic stimulation (TMS) may yield synergistic benefits, as TMS enhances neural plasticity while GIM promotes emotional and cognitive restructuring (Hernández-Sauret et al., 2024). Additionally, technological innovations—such as virtual reality (VR) for immersive therapeutic environments, artificial intelligence (AI) for dynamic music personalization, and digital platforms for remote delivery—could significantly improve GIM’s scalability, accessibility, and treatment adherence (Lin et al., 2020; Bakas et al., 2021; Jiao, 2025).

Additionally, it is worth mentioning the highly debated “Mozart Effect” (McLachlan, 1993). This theory posits that listening to certain types of classical music (such as compositions by Mozart) may temporarily enhance cognitive performance, particularly in spatial reasoning. Although its underlying mechanisms have not been fully confirmed, this effect may be related to the emotional improvement, neural activation, and attention regulation induced by music, thereby indirectly influencing cognitive and brain function (Giannouli, 2017). While research on the “Mozart Effect” has primarily focused on healthy populations and cognitive tasks, the underlying “music–emotion–cognition” associative mechanism provides a valuable supplementary perspective for understanding how GIM regulates cognitive function through emotional pathways. Future studies could further explore the similarities and differences in neural mechanisms and clinical outcomes between such “structural music listening” and depth-oriented intervention methods, thereby enriching the application paradigms of music in the field of mental health.

It is noteworthy that the influence of personal preference on outcomes is closely related to the placebo effect. A study on the Mozart Effect revealed that participants with a strong preference for Mozart’s music performed significantly better on cognitive tasks compared to others, even when there was no evidence indicating that one piece of music objectively outperformed another (Giannouli et al., 2010). This finding suggests that patients’ inherent beliefs or preferences for specific interventions can influence treatment outcomes through expectations and levels of engagement. Therefore, it is crucial to assess and consider patients’ preferences for music and therapy. Doing so not only helps distinguish the specific neurobiological effects of interventions from non-specific placebo effects but also facilitates the development of personalized treatment plans, potentially amplifying overall therapeutic outcomes.

In summary, this review introduces an integrative neural circuit-based framework for GIM, synthesizing its multimodal mechanisms and clinical benefits. With ongoing scientific exploration and technological integration, GIM holds substantial potential to evolve into a more personalized, scalable, and evidence-based therapeutic approach for MDs and related psychiatric disorders.
